# Hemoglobin Wayne: A Rare Variant That Can Cause Falsely Elevated Hemoglobin A1c

**DOI:** 10.7759/cureus.26559

**Published:** 2022-07-04

**Authors:** Xue Ao, Nagapratap Ganta, Suhrim Choe, Prachi Patel, James Turro, Pramil Cheriyath

**Affiliations:** 1 Internal Medicine, Hackensack Meridian Health Ocean Medical Center, Brick, USA; 2 Internal Medicine, Rajarshee Chhatrapati Shahu Maharaj Government Medical College, Kolhapur, IND

**Keywords:** diabetes mellitus dm, glycated hemoglobin (hba1c), rare variant, elevated hba1c, hemoglobin wayne, hemoglobin variant

## Abstract

Hemoglobin A1c (HbA1c) can be unreliable (falsely elevated or lowered) in certain conditions, including hemoglobinopathies, anemia, lead poisoning, chronic alcoholism, and opioid use. Hemoglobin Wayne is a rare variant of hemoglobin (Hgb) that can also result in a false elevation of HbA1c. Hence, clinicians should be aware of these underlying causes before diagnosing and treating diabetes mellitus to avoid unexpected consequences. We are reporting a case of falsely elevated HbA1c in a female in her early 60s due to a rare variant of Hgb called hemoglobin Wayne. The patient presented with a consistently elevated HbA1c ranging from 10.3% to 10.7% for two years, which did not correlate with her fasting blood glucose levels ranging between 80 and 100. The continuous glucose monitoring (CGM) profile was also within the normal range. The hemoglobin electrophoresis technique was used to confirm the diagnosis of hemoglobin Wayne in this patient and the initial treatment of metformin was discontinued upon confirmation.

## Introduction

Diabetes mellitus (DM) can be diagnosed using two consecutive measures of glycated hemoglobin (A1c) ≥ 6.5% (48 mmol/mol), according to the World Health Organization and the American Diabetes Association [1]. It is well established that some hemoglobin (Hgb) variations can skew A1c results in a variety of ways [2]. There are around 1000 distinct Hgb variations, the majority of which are clinically silent. Such polymorphisms can now be regularly recognized because of the widespread use of chromatographic techniques for hemoglobin A1c (HbA1c) detection. High-performance liquid chromatography (HPLC) (VARIANT II, Bio-Rad, Hercules, CA), alkaline and acid electrophoresis (HYDRASYS 2, Sebia, Lisses, France), capillary zone electrophoresis (CAPILLARYS 2, Sebia, Lisses, France), and DNA sequencing are used to investigate hemoglobinopathy [3]. A frameshift mutation in the HBA2 gene causes hemoglobin Wayne, an uncommon alpha chain Hgb variant [4]. We are reporting a case of falsely elevated HbA1c in a 63-year-old Caucasian female because of the presence of hemoglobin Wayne, a rare Hgb variant.

## Case presentation

A 63-year-old Caucasian female patient presented to the clinic for a follow-up visit. She was diagnosed with type 2 diabetes mellitus (T2DM) in an outside clinic because of persistently elevated HbA1c of 10.3% to 10.7% since January 2020. Her A1c was initially 5.8% three years ago (2018). She was started on metformin therapy by her previous primary care physician. However, the patient did not experience any symptoms or complications secondary to diabetes, such as foot numbness, acute vision changes, chest pain, headache, dizziness, change in vision, shortness of breath, belly pain, nausea, vomiting, polyuria, or polydipsia. The patient's past medical history included monoclonal gammopathy, rheumatoid arthritis, osteoporosis, and dry eyes and mouth secondary to Sjögren's syndrome. Current medications included etanercept subcutaneous injection once a week, ibandronate 150 mg by mouth once a month, cevimeline 30 mg capsule three times a day, and ezetimibe 10 mg daily. Family history was significant for diabetes mellitus in the mother and heart disease in the father. The patient denied any history of smoking, alcohol, or recreational drug use. As part of her diabetes management, the patient was told to log her blood glucose levels regularly. A discrepancy was found between the A1c levels and the blood glucose readings. The patient's blood glucose levels persistently ranged between the 80s and 100s. The initial treatment of metformin 500 mg once daily when HbA1c was elevated was later discontinued. Continuous glucose monitoring (CGM) profile was ordered and the results were within the normal range. These blood glucose levels did not correlate with consistently elevated A1c levels of >10%. Insulin therapy was not initiated initially due to this discrepancy in the findings of blood glucose levels and HbA1c. The possibility of a confounding factor was considered. The fructosamine assay was reportedly within the normal range. Hemoglobin electrophoresis was ordered, and hemoglobin pattern and concentration were consistent with an alpha variant, as seen in hemoglobin Wayne. The results showed hemoglobin Wayne variant at 13.2% (0%), Hgb A at 84.5% (96.4-98.8%), and Hgb A2 at 2.3% (1.8-3.2%) (Table 1). The patient was sent home without further treatment based on the confirmation that the elevated HbA1c was not truly raised.

**Table 1:** Results of hemoglobin electrophoresis reflex to HPLC fractionation Hgb = hemoglobin; HPLC = high-performance liquid chromatography.

	Measured value	Normal range
Hgb F	0.0%	0.0-2.0%
Hgb A	84.5% (low)	96.4-98.8%
Hgb A2	2.3%	1.8-3.2%
Hgb S	0.0%	0.0%
Hgb C	0.0%	0.0%
Hgb E	0.0%	0.0%
Hgb variant	13.2% (high)	0.0%

## Discussion

Although hemoglobin A1c is commonly used to diagnose diabetes and monitor the effects of medications in individuals with diabetes, it can be troublesome in patients with hemoglobinopathies or disorders that affect red cell turnovers, such as hemolytic anemia, blood transfusions, or severe blood loss [1]. Reduced erythropoiesis can generate an erroneously elevated HbA1c (e.g. iron or vitamin B12 deficiency). Alcoholism, lower intra-erythrocyte pH, opioids, lead poisoning, salicylates, and chronic renal failure are mechanisms that directly affect glycation. Hgb variants or medicines such as high doses of aspirin or persistent opiate use might cause assays to fail, resulting in artificially elevated HbA1c levels [5]. When assessed by electrophoresis, vitamin C may raise A1c levels, but when analyzed by chromatography, it may lower them. This could be due to a competitive mechanism inhibiting glycosylation. Although this benefit was detected with the administration of one gram of vitamin C daily for three months, a dose regularly taken by many individuals, the clinical value of this study is unknown because it was undertaken in patients without diabetes [6].

Hemoglobin A0 (α2β2) is the primary hemoglobin component in human red blood cell hemolysate, accounting for 90% of the total protein. Several glycosylated minor hemoglobin variations can be isolated from the primary component hemoglobin A0 using chromatography, including hemoglobin A1a1, A1a2, A1b, and A1c. In human red blood cells, A1c is the most abundant minor component, accounting for around 5% of the total. This hemoglobin was found to be two to threefold higher in diabetic people. Indeed, an increase in A1c indicates a 120-day increase in mean plasma glucose concentrations (the average erythrocyte lifespan). Some A1c assay methods are hampered by hemoglobinopathies, which affect red blood cell survival or hemoglobin glycation [1].

A frameshift mutation in the HBA2 gene causes hemoglobin Wayne, an alpha chain Hgb variation (HBA2:c.420del) [3]. It was initially discovered in a seven-year-old child with Fanconi anemia in 1976. Because information is scarce regarding this rare mutation, the prevalence of the hemoglobin Wayne characteristic is unknown [4]. Single amino acid substitutions cause the majority of hemoglobin variants. Deamination of the hemoglobin Wayne protein occurs at asparagine 139, resulting in two hemoglobin Wayne molecules: hemoglobin Wayne I (asparagine as residue 139) and hemoglobin Wayne II (aspartic acid as residue 139) [1]. Most heterogeneous hemoglobinopathies are benign and clinically silent, and if not considered as the reason for falsely increased A1c, they can go unnoticed, leading to misdiagnosis of DM. When there are disparities in A1c and blood glucose levels, several studies suggest that the presence of hemoglobin variations should be evaluated as a differential [1,2].

Immunoassays, ion exchange HPLC, boronate affinity HPLC, and enzymatic assays are the four most popular methods for measuring A1c. Electrophoresis and HPLC analyses of hemoglobin are the most often utilized procedures to aid in the identification of hemoglobinopathies. To distinguish HbA1c molecules from other hemoglobin molecules, a chromatography-based assay uses an HPLC apparatus and ion exchange or affinity column, and the result is calculated by dividing the HbA1c peak area by the total hemoglobin peak area [7]. For our patient, A1c was measured by cation exchange HPLC system using the Bio-Rad VARIANT II Turbo analyzer.

According to studies, hemoglobin Wayne changes hemoglobin charge and confounds HbA1c by interfering with the commonly used cation exchange HPLC measurement. Hence, other methods such as boronate affinity assay, molecular analysis, and DNA sequencing can be used, as they are not affected by hemoglobinopathies [8]. However, these methods may miss some hemoglobin variations, necessitating extra testing [1]. Boronate affinity techniques utilize m-aminophenylboronic acid, which reacts selectively with the cis-diol groups of glucose coupled to Hgb. This approach detects total glycated Hgb, which includes HbA1c and Hgb glycated at other sites, with the least amount of interference from Hgb variations and derivatives. Methods are categorized by kind, as well as whether the result is artificially enhanced or decreased due to the existence of a hemoglobin variation. Laboratorians should be aware of their method's limitations in terms of these interferences. In the year 2000, an estimated 12 million people with diabetes mellitus had a hemoglobin variant. This figure is predicted to rise to 26 million by 2030 [9].

## Conclusions

There are several learning points to focus on in this case report. It is crucial to demonstrate an appropriate correlation between HbA1c and blood glucose levels before diagnosing diabetes mellitus. Physicians should always be vigilant and look for underlying causes of falsely elevated HbA1c if there is no correlation between elevated HbA1c and blood glucose levels, which is supported by CGM data. If the need arises, then consider changing the methods to determine HbA1c to boronate affinity techniques, which have the least amount of interference from Hgb variations and derivatives compared to electrophoresis or ion exchange HPLC. Unnecessary treatment for falsely elevated HbA1c can lead to severe consequences like hypoglycemia and bradycardia, to name a few. This holds good for low HbA1c values also; if there are low values of HbA1c and discordant with blood sugar estimation, interference by variants should be considered. Persistently low HbA1c in the presence of high blood sugar is equally prone to treatment errors as under-treatment based on low HbA1c can harm the patient. Therefore, both abnormally low and high HbA1c values need to be looked at for Hgb variants.
